# Cocaine exposure impairs multineage hematopoiesis of human hematopoietic progenitor cells mediated by the sigma-1 receptor

**DOI:** 10.1038/srep08670

**Published:** 2015-03-02

**Authors:** Christopher C. Nixon, Brandon H. Schwartz, Dhaval Dixit, Jerome A. Zack, Dimitrios N. Vatakis

**Affiliations:** 1 Department of Microbiology, Immunology, & Molecular Genetics, UCLA; 2 Department of Integrative Biology & Physiology, UCLA; 3 Department of Hematology/Oncology, Division of Medicine, David Geffen School of Medicine, UCLA; 4 UCLA AIDS Institute, David Geffen School of Medicine, UCLA

## Abstract

Prenatal exposure to cocaine is a significant source of fetal and neonatal developmental defects. While cocaine associated neurological and cardiac pathologies are well-documented, it is apparent that cocaine use has far more diverse physiological effects. It is known that in some cell types, the sigma-1 receptor mediates many of cocaine's cellular effects. Here we present a novel and concise investigation into the mechanism that underlies cocaine associated hematopoietic pathology. Indeed, this is the first examination of the effects of cocaine on hematopoiesis. We show that cocaine impairs multilineage hematopoiesis from human progenitors from multiple donors and tissue types. We go on to present the first demonstration of the expression of the sigma-1 receptor in human CD34 + human hematopoietic stem/progenitor cells. Furthermore, we demonstrate that these cocaine-induced hematopoietic defects can be reversed through sigma-1 receptor blockade.

World annual cocaine use currently stands at around 600 metric tons, with Americans consuming roughly half and Europeans, about a quarter and thus represents a significant public health concern. Specifically, prenatal exposure to drugs of abuse is a significant source of fetal developmental pathology^1^. The majority of research on the health impact of cocaine use on fetal health has been focused on neurological^2^,^3^,^4^ and cardiac effects^5^,^6^ and these are well documented. In contrast, little is known about the effects of cocaine on hematopoiesis.

Prenatal cocaine exposure clearly impacts development. Cocaine is able to cross the placenta and the blood-brain barrier allowing it to impact the fetus^7^. Structural abnormalities are evident in the brains^8^ and hearts^9^ of neonates exposed to cocaine in utero. However, it is not known if this is the result exclusively of direct toxicity to cells of the developing nervous and/or cardiac systems or other effects of cocaine use. Low-birth weight has also been documented in neonates born to mothers who use cocaine^10^,^11^,^12^.

Cocaine exposure is known to influence the function of immune cells. Cocaine can increase T-cell apoptosis, alter immune related miRNA profiles, change immune activation state, and impair lymphocyte proliferation^13^,^14^,^15^,^16^,^17^. However, little has been studied in terms of how drugs of abuse impact hematopoiesis. There is a single clinical report of severe aplastic anemia following extreme chronic 3,4-methylenedioxy-N-methylamphetamine (MDMA) use^18^ and there are a handful of reports addressing the effects of marijuana on blood cell development^19^. Reports of hematopoietic disregulation due to cocaine use are few but do support the hypothesis that cocaine disrupts normal blood cell homeostasis^20^,^21^. Cocaine exerts its effects at a cellular level in-part through the sigma-1 receptor (S1R)^22^,^23^,^24^,^25^. The S1R is an endoplasmic reticulum molecular chaperone expressed in a variety of cell types. Depending on the context, the S1R is able to either inhibit or potentiate a number of different ion channels. Furthermore, the S1R has been implicated in a number of diseases including HIV, cancer, depression and addiction^22^,^23^.

Previously, we demonstrated that quiescent T cell susceptibility to HIV infection can be modulated by cocaine, in part through its interactions with the S1R^22^. Here, we report the singular finding that the developmental potential of human hematopoietic progenitor cells that are exposed to nontoxic levels of cocaine was severely depressed. Interestingly, we found that S1R blockade relieved the impairment and allowed for normal hematopoiesis to proceed. Thus, our data suggest that stimulant exposure can impact human hematopoiesis and that the S1R may play a role in this developmental process, beyond substance abuse.

## Results

### Nontoxic levels of cocaine exposure depresses multilineage hematopoiesis from human CD34 + HSPC

**Figure 1:**
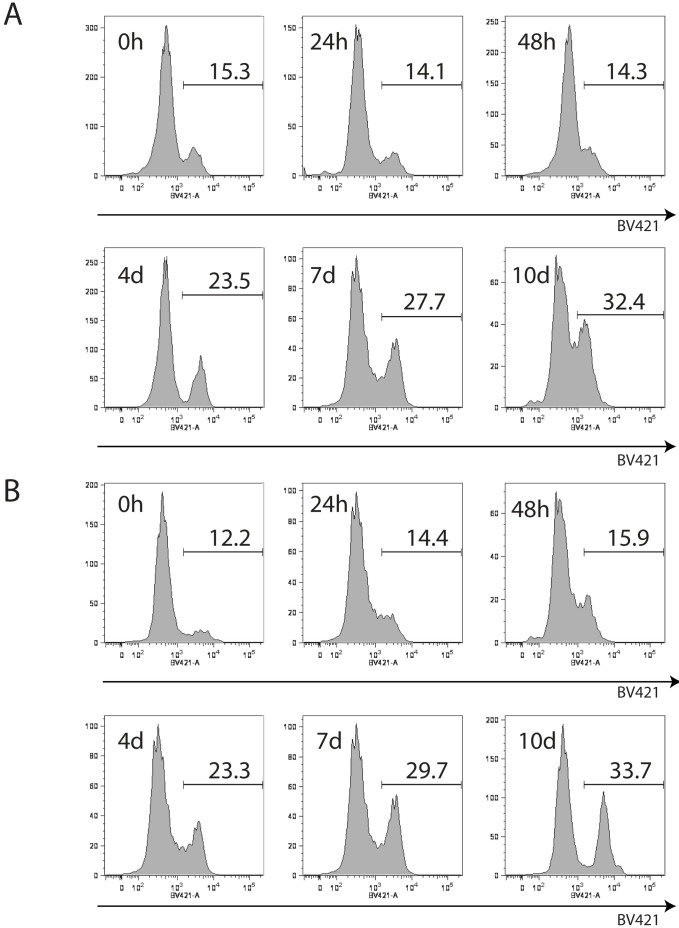
Cocaine is not immediately toxic to HSPCs. Representative live/dead dye exclusion flow cytometry viability assay of (A) cocaine treated and (B) untreated HSPC in culture for ten days. For each, top left panel: 0 hrs, top middle panel: 24 hrs, top right panel: 48 hours, bottom left panel: 5 days, bottom middle panel: 7 days, bottom right panel: 10 days.

We first sought to determine if exposure to physiologically relevant concentrations of cocaine is toxic to human hematopoietic stem progenitor cells (HSPCs). Cocaine treatment levels and exposure time were used based on previous studies that determined on the basis of physiological effect on T cells without any toxicity risks^22^,^26^. CD34 + progenitors from fetal liver (FL) were incubated at varying timepoints with cocaine. Following treatment, a portion of the cells from both cocaine exposed and untreated cultures were examined by flow cytometry for cell death. Representative flow cytometry plots indicate no apparent differences in cell viability comparing untreated and cocaine treated cells at 24 and 48 hours post-exposure (Fig. 1a,b). Prior to cocaine exposure, cultures were approximately 85% viable while they were approximately 86% viable at 24 and 48 hours post drug exposure. Cultures were further examined for up to 10 days following exposure and monitored for evidence of toxicity(Fig. 1a,b). There were no evident differences in the viability of cultures that were exposed to cocaine compared to those that were not. These data indicate that cocaine is nontoxic to HSPCs in culture at a concentration of 10^−8^ M.

**Figure 2:**
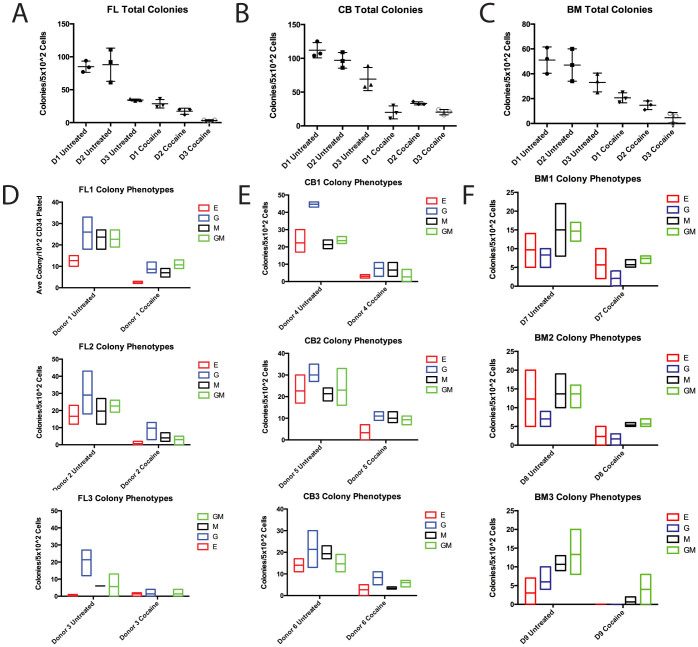
Nontoxic cocaine exposure impairs multilineage hematopoiesis. (A) Methylcellulose colony forming assay for untreated and cocaine treated HSPC derived from three different FL donors. Each mark represents the score on a sinlge plate, and the bar marks the mean. (B) Methylcellulose colony forming assay for untreated and cocaine treated HSPC derived from three different CB donors. Each mark represents the score on a sinlge plate, and the bar marks the mean. (C) Methylcellulose colony forming assay for untreated and cocaine treated HSPC derived from three different BM donors. Each mark represents the score on a sinlge plate, and the bar marks the mean. (D) Phenotypic analysis of colonies that developed from FL derived HSPCs treated with cocaine. (E) Phenotypic analysis of colonies that developed from CB derived HSPCs treated with cocaine. (F) Phenotypic analysis of colonies that developed from BM derived HSPCs treated with cocaine. Red boxes/E erythroid colonies, blue boxes/G granulocyte colonies, black boxes/M macrophage colonies, green boxes/GM mixed granolocyte/macrophage colonies.

Since cocaine had no apparent impact on cell viability, we then addresed whether cocaine exposure alters the hematopoietic potential of HSPCs derived from either FL, cord blood (CB), or adult bone marrow (BM). A portion of the cells from cocaine treated and untreated cultures were plated in complete methylcellulose and analyzed two weeks later for the development of hematopoietic colonies. In plates derived from HSPCs treated with cocaine, total colony number was severely reduced compared to untreated HSPCs (Fig. 2a,2b,2c). In FL derived cultures from three separate donors, cocaine treated cells gave rise to an average of 23 +/− 3 total colonies while untreated cells gave rise to an average of 87 +/− 17 total colonies. This difference is statistically significant by two tailed t-test with a P value <0.001. In CB derived cultures from three separate donors, cocaine treated cells gave rise to an average of 25 +/− 3 total colonies while untreated cells gave rise to an average of 93 +/− 17 total cells. This difference is statistically significant by two-tailed t-test with a p value <0.001. In BM derived cultures from three individual donors, cocaine treated progenitors gave rise to an average of 13 +/− 3 total colonies while untreated progenitors gave rise to an average of 44 +/− 5 total colonies. This difference is statistically significant by two-tailed t-test with a p value <0.001.

When colonies are analyzed by phenotype, development of all lineages are severely impaired in cultures derived from FL (fig. 2d), CB (fig. 2e) and BM (fig. 2e). Indeed, Table 1 shows that in almost every case derived from FL and CB, each type of colony from each donor, from both source tissue types, HSPCs that have been exposed to nonlethal levels of cocaine are impaired for development of all cell types. There is no apparent pattern of donor/colony types in which development is not impaired in a statistically significant fashion following drug exposure. All erythroid colonies from all but donor 3 showed that difference between the number of colonies that develop from untreated compared to cocaine treated progenitors. It is difficult to interpret the colony numbers of both the erythroid and the granulocyte/macrophage mixed cultures due to the fact that the numbers of these colony types in untreated cultures were quite low. However, there is strong significance in the differences in total colony number from this donor. BM derived cultures show similar results. While there were no differences in erythroid colonies that developed from cocaine treated compared to untreated progenitors, the development of almost all other myeloid colonies from all donors were suppressed in cultures derived from treated progenitors. Overall, these data indicate that cocaine exposure suppresses myeloid development from HSPCs derived from multiple tissues and types.

### The sigma-1 receptor is expressed in human CD34+ HSPCs

**Figure 3:**
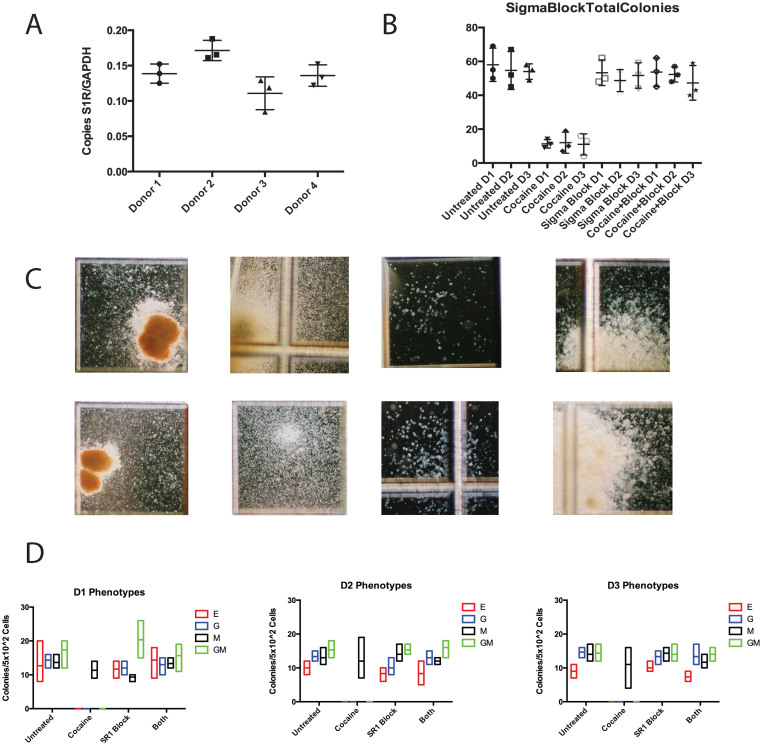
The sigma-1 receptor is expressed in HSPCs and mediates cocaine's hematosuppressive effects. (A) RT-PCR analysis of sigma-1 receptor in FL derived HSPCs from four separate donors. S1R expression is graphed relative to GAPDH expression and the average of three replicate reactions is plotted. (B) Methylcellulose colony forming assays of FL derived HSPCs that were either untreated, cocaine treated, S1R block treated, and cocaine + block treated. Each mark represents the score on a single plate, and the bar marks the mean. (C) Representative images of hematopoietic colonies that developed from S1R-block treated (top) or untreated (bottom) HSPC. (D) Phenotypic analysis of colonies that developed from HSPCs that were either untreated, cocaine treated, S1R block treated, and cocaine + block treated. Red boxes/E erythroid colonies, blue boxes/G granulocyte colonies, black boxes/M macrophage colonies, green boxes/GM mixed granolocyte/macrophage colonies.

As previous work has implicated the sigma-1 receptor in mediating the cellular effects of cocaine^22^,^26^, we sought to determine if this receptor is present in human HSPCs. RT-PCR anaylsis for the sigma-1 receptor transcript in FL derived HSPCs from four separate donors indicate that it is indeed expressed in blood progenitors (Fig. 3a). The average of three RT-PCR reactions from donor 1 indicated 0.14 +/− 0.0078 copies of S1R per copy of GAPDH, while it was 0.17 +/− 0.0083 for donor 2, 0.11 +/− 0.013 for donor 3, and 0.14 +/− 0.0088 for donor 4.

### S1R blockade alleviates cocaine-induced suppression of hematopoiesis

To elucidate the mechanism by which cocaine suppresses hematopoiesis, we expanded our cocaine exposure experiments to include blockade of the sigma-1 receptor. FL derived HSPCs from three separate donors were either left untreated or treated with cocaine, S1R blockade, or both for 48 hours and then plated in complete methylcellulose. Two weeks later, plates were scored for hematopoietic colony development. The total number of colonies that developed in cultures derived from cells that were treated with either S1R blockade or cocaine and S1R blockade were indiscernible while cultures that were treated with cocaine alone show significantly reduced total colony numbers (Fig. 3b). In this set of experiments, untreated HSPCs from all three donors gave rise to an average of 56 +/− 13 colonies per plate while those exposed to cocaine gave rise to only 12 +/− 2 colonies per plate. HSPCs that were treated with only the receptor block gave rise to 51 +/− 12 colonies and HSPCs that were treated with receptor block and cocaine gave rise to 51 +/− 13 colonies per plate. There were no significant differences in the total colony number that developed from untreated, receptor block treated, or receptor block and cocaine treated HSPCs.

Phenotypic analysis indicates that neither treatment with S1R block or S1R block and cocaine appears to alter hematopoietic lineage commitment (Fig. 3d). To confirm that treatment with the receptor block is not toxic to developing hematopoietic colonies, individual colonies from each of the treatment conditions were examined by microscopy (Fig. 3c). Table 2 shows that only cocaine exposure results in a statistically significant loss of developmental potential. Interestingly, macrophages were the only lineage that was not impaired with cocaine treatment. Granulocyte, macrophage, and mixed granulocyte/macrophage colonies were clearly suppressed by cocaine exposure of HSPCs from all three donors. There were no statistically significant differences in the colony forming potential between untreated HSPC and those treated with just S1R blockade or both S1R blockade and cocaine. Together these data clearly show that the S1R can mediate changes in human hematopoiesis.

## Discussion

In conclusion, our studies strongly demonstrate that cocaine exposure has far reaching effects on human development and is detrimental to early and intermediate stages of human hematopoiesis. We show that in HSPCs derived from both fetal liver and cord blood, cocaine exposure results in depressed hematopoietic potential as determined by colony forming assays. Moreover, we show that blockade of the sigma-1 receptor restores normal hematopoietic potential. Together these data establish the novel concept that cocaine exposure modulates human hematopoiesis.

It was previously reported that acute cocaine exposure induced terminal erythrocytosis as measured by hematocrit and reticulocyte count^18^. While this may seem to contradict the data presented here, it is important to note that erythroid colonies that develop in methylcellulose are not composed of terminally differentiated red blood cells but a mixture of intermediate-late erythroid progenitors. The investigators reported an increase in hematocrit but not reticulocyte count which would indicate an increase in terminally differentiated erythrocytes but not one in the production and/or release of immature red blood cells. Furthermore, the study consisted only of cocaine users who reported to the emergency department with drug associated acute chest pain. Therefore, we do not believe this represents a accurate picture of cocaine exposure.

Interestingly, macrophages seemed unaffected by cocaine exposure in the last set of experiments. This suggests the possibility that a macrophage precursor may be tolerant to the effects of cocaine exposure and demands further study. As this was not observed in the first set of experiments, more detailed analysis will be required. It is important to remember that the CD34 + population is composed of diverse phenotypes and each sample is unique it its distribution of these. Taken together, these points clearly indicate that each subpopulation of HSPCs should be studied under the same conditions.

This work opens the door to further study of the impact of drugs of abuse on human hematopoiesis. An early study indicated that mice deficient in the mu-opiod receptor, which mediates the cellular effects of a number of drugs of abuse, showed increased developmental potential and were in a much higher cycling state of HSPC relative to their wild-type counterparts^27^.

As this may potentially have downstream effects on the immune system of children exposed to cocaine in utero, S1R blockade may represent a potential protective treatment for women at high risk for drug abuse during pregnancy. Moreover, it is possible that some of the impaired development that results from in utero cocaine expose could be due to impaired blood development.

## Methods

### Ethics statement

Peripheral blood mononulear cells and cord blood were obtained at the University of California, Los Angeles in accordance with UCLA Institutional Review Board (IRB) approved protocols under written informed consent using an IRB-approved written consent form by the UCLA/CFAR Virology Laboratory and was distributed for this study without personal identifying information. Human fetal tissue was purchased from the UCLA/CFAR Gene Therapy Core, was obtained without identifying information and did not require IRB approval for use. All experiments and agents used were carried out and used in accordance with the UCLA Institutional Biosafety Committee (IBC).

### Isolation and culture of human hematopoietic stem cells

Mononuclear cells were obtained from the buffy coats of either processed fetal liver or cord blood or adult bone marrow (Allcells, Alameda, CA) following Ficoll-Hypaqe density gradient separation. CD34 + HSPCs were sorted from buffy coats by MACS using CD34 + magnetic beads (Miltenyi Biotec, Auburn, CA, USA) and LS columns (Miltenyi Biotec) following manufacturer's protocol. Cells were cultured in StemSPAN™ serum-free media (Stem Cell Technologies) supplemented with stem cell factor (Life Technologies, Grand Island, NY, USA), thrombopoietin (Life Technologies), IGF-BP2 (Peprotech, Rocky Hill, NJ, USA), and Flt3-ligand (Life Technologies), all at a concentration of 100 ng/mL.

### Cocaine treatment and sigma-1 receptor blockade

Cocaine was added to cultures at a concentration of 10^−8^ M. The sigma-1 receptor blockade BD1047 (Sigma-Aldrich, St. Louis, MO, USA) was added to cultures at a concentration of 10^−6^ M. Both remained in culture for forty eight hours until cells were plated in methylcellulose.

### Colony forming assays

Cells were plated in Methocult™ H4435 enriched methylcellulose (Stem Cell Technologies, Vancouver, BC) at a concentration of 500/mL and cultured in 35 mm grid plates, 1 mL per plate, in triplicate per condition, for two weeks in a 37°C 5% CO_2_ incubator. Hematopoietic colonies were scored in a blinded fashion. Colonies were counted on a Nikon Eclipse TE300 microscope at 400x magnification.

### Real-time RT-PCR

RNA was isolated from cells using the RNeasy kit (Qiagen) following the manufacturer's protocol. PCR reactions were performed using the One Step RT-PCR Kit for Probes (Biorad, Hercules, CA, USA) and FAM labeled primer-probsets for SIR and GAPDH transcripts manufactured by Advanced Biosystems Inc. Twenty μl reactions were prepared for each sample with 5 μl of target RNA. All samples were run in triplicate. Samples were analyzed along with standards of known concentration to allow for quantification. All reactions were carried out on an IQCycler thermalcycler (Biorad). Cycling conditions were: 1 cycle of reverse transcription for 10 min at 50° C, one denaturation cycle for 3 min at 95°C, followed by 40 amplification cycles. Analysis was performed with iQ5 software (Biorad).

### Flow cytometry

The Live/Dead® Aqua dye (Life Technologies) was used following manufacturers protocol to determine viability in HSPC cultures. Briefly, 1 × 10^6^ cells were harvested from untreated and cocaine treated cultures at the indicated timepoints and washed twice and then resuspended in 1 mL of phosphate-buffered saline (PBS). One uL of dye was added to each sample, mixed, and then incubated in the dark for 30 minutes. Cells were then washed and fixed for 15 minutes in PBS-37% formaldehyde. Fixed cells were maintained in 500 uL PBS-37% formaldehyde at 4°C in the dark until flow cytometry analysis. All stained cells were analyzed together on an LSRFortessa™ (BD Biosciences, San Jose, CA, USA) flow cytometer.

### Statistics

All statistical analysis was performed with Graphpad Prism software (La Jolla, CA, USA). P values < 0.05 were considered significant.

## Author Contributions

C.C.N., J.A.Z. and D.N.V. wrote the paper and designed the experiments. C.C.N., B.H.S. and D.D. performed the experiments.

## Figures and Tables

**Table 1:** Hematopoietic colonies derived from untreated or cocaine exposed CD34 + cells from either fetal liver, cord blood, or adult bone marrow. Donors 1–3 represent fetal liver, donors 4–6 represent cord blood, and donors 7–9 represent adult bone marrow. Values are the Mean and SEM. E, erythroid, M, macrophage, G, granulocyte, GM, mixed granulocyte-macrophage colonies

	E	P value	M	P value	G	P value	GM	P value	Total	P value
Donor 1	12.67 +/− 1.453	0.0023	23.67 +/− 6.848	0.0056	26.00 +/− 4.359	0.0206	22.67 +/− 2.333	0.0102	85.00 +/− 4.933	0.0008
Cocaine	2.333 +/− 0.333		7.00 +/− 1.155		8.667 +/− 1.667		10.67 +/− 1.202		26.67 +/− 3.712	
Donor 2	16.67 +/− 3.283	0.0088	19.67 +/− 4.333	0.027	29.00 +/− 7.371	0.0752	20.67 +/− 2.028	0.0015	88.00 +/− 14.57	0.0088
Cocaine	0.6667 +/− 0.6667		4.000 +/− 1.528		9.667 +/− 3.333		3.000 +/− 1.528		17.33 +/− 2.667	
Donor 3	1.333 +/− 0.6667	0.4216	6.000 +/− 0.5774	0.0005	21.33 +/− 4.702	0.015	5.667 +/− 3.844	3.469	34.33 +/− 8.0819	0.001
Cocaine	0.6667 +/− 0.3333		0.0 +/− 0.0		1.333 +/− 1.333		1.333 +/− 1.333		3.333 +/− 0.6667	
Donor 4	22.33 +/− 3.390	0.0082	21.33 +/− 4.153	0.0059	44.67 +/− 10.88	0.001	23.67 +/− 2.102	0.001	112.0 +/− 6.557	0.0004
Cocaine	3.000 +/− 0.5774		6.667 +/− 2.333		7.667 +/− 2.404		2.667 +/− 2.186		20.0 +/− 5.508	
Donor 5	22.67 +/− 8.344	0.0113	21.33 +/− 7.164	0.083	30.00 +/− 5.217	0.0024	23.00 +/− 5.132	0.065	97.00 +/− 6.658	0.0007
Cocaine	3.333 +/− 2028		10.00 +/− 1.528		11.00 +/− 1.155		9.333 +/− 1.202		33.33 +/− 1.333	
Donor 6	14.00 +/− 1.732	0.0074	19.33 +/− 1.856	0.0011	21.33 +/− 4.910	0.0674	14.67 +/− 2.333	0.0269	69.33 +/− 9.871	0.0085
Cocaine	2.667 +/− 1.453		3.333 +/− 0.3333		8.333 +/− 1.764		6.000 +/− 1.000		20.33 +/− 2.333	
Donor 7	9.667 +/− 2.603	0.3164	15.00 +/− 4.041	0.0849	8.333 +/− 1.667	0.0354	14.67 +/− 1.453	0.0101	51.00 +/− 6.083	0.0096
Cocaine	5.667 +/− 2.333		5.667 +/− 0.6667		2.000 +/− 1.155		7.333 +/− 0.6667		20.67 +/− 2.333	
Donor 8	12.33 +/− 4.333	0.0939	13.67 +/− 2.728	0.0387	7.00 +/− 1.155	0.0214	13.67 +/− 1.856	0.0154	47.00 +/− 7.506	0.0142
Cocaine	2.333 +/− 1.453		5.333 +/− 0.333		1.667 +/− 0.882		5.667 +/− 0.6667		14.67 +/− 2.028	
Donor 9	3.00 +/− 2.082	0.223	10.67 +/− 1.202	0.002	6.00 +/− 2.00	0.04	13.33 +/− 3.528	0.091	33.00 +/− 4.359	0.005
Cocaine	0.00 +/− 0.00		0.667 +/− 0.667		0.00 +/− 0.00		4.00 +/− 2.309		4.667 +/− 2.404	

**Table 2:** Hematopoietic colonies derived from either untreated, cocaine, SR1 block, or cocaine and SR1 block exposed CD34 + cells that had been isolated from fetal liver. Values are the Mean and SEM. E, erythroid, M, macrophage, G, granulocyte, GM, mixed granulocyte-macrophage colonies

	E	P value	M	P value	G	P value	GM	P value
Donor 1	12.67 +/− 3.712		13.67 +/− 2.333		14.33 +/− 2.906		17.33 +/− 6.360	
Cocaine	0.0 +/− 0.0	0.027	11.33 +/− 2.603	0.541	0.0 +/− 0.0	0.0079	0.0 +/− 0.0	0.0527
S1R Block	11.67 +/− 1.453	0.8143	9.333 +/− 2.963	0.3416	12.00 +/− 2.517	0.5766	20.33 +/− 3.180	0.6948
Both	14.33 +/− 2.728	0.7358	13.33 +/− 2.603	0.9286	13.00 +/− 2.082	0.7281	15.67 +/− 2.404	0.8184
Donor 2	10 +/− 2.887		13.00 +/− 2.646		13.33 +/− 2.603		15.33 +/− 3.756	
Cocaine	0.0 +/− 0.0	0.0257	12.00 +/− 3.606	0.834	0.0 +/− 0.0	0.0069	0.0 +/− 0.0	0.015
S1R Block	8.333 +/− 2.333	0.6767	14.00 +/− 3.215	0.822	10.00 +/− 3.125	0.4655	15.33 +/− 0.8819	0.9999
Both	8.333 +/− 2.028	0.6612	12.00 +/− 3.215	0.822	13.00 +/− 2.309	0.9283	16.00 +/− 3.606	0.9043
Donor 3	9.00 +/− 2.887		14.00 +/− 3.215		14.67 +/− 3.180		14.33 +/− 2.603	
Cocaine	0.0 +/− 0.0	0.0356	11.00 +/− 3.606	0.5682	0.0 +/− 0.0	0.0099	0.0 +/− 0.0	0.0053
S1R Block	10.00 +/− 3.215	0.8283	14.33 +/− 4.055	0.8722	13.33 +/− 2.906	0.7724	14.00 +/− 3.786	0.9456
Both	7.333 +/− 2.028	0.6612	11.67 +/− 3.383	0.6433	13.33 +/− 4.096	0.8098	14.00 +/− 1.555	0.8662
